# 2737. Real World Evaluation of Antifungal Prophylaxis in Allogeneic Hematopoietic Stem Cell Transplant (aHSCT) Patients, Is Routine Antifungal Prophylaxis Needed?

**DOI:** 10.1093/ofid/ofad500.2348

**Published:** 2023-11-27

**Authors:** Sarah Perreault, Molly Schiffer, Heidi Roeder, Dayna McManus, Jeffrey E Topal

**Affiliations:** Yale New Haven Hospital, New Haven, Connecticut; Yale New Haven Health, New Haven, Connecticut; Yale New Haven Health, New Haven, Connecticut; Yale New Haven Hospital, New Haven, Connecticut; Yale New Haven Hospital Yale University School of Medicine, New Haven, Connecticut

## Abstract

**Background:**

Invasive fungal infections (IFI) are a known complication in aHSCT patients. Risk factors for IFI post aHSCT include history of IFI, high intensity conditioning regimens, prolonged neutropenia, and degree of donor matching. Guidelines recommend anti-*Candida* prophylaxis in low risk patients and anti-mold prophylaxis in high risk patients through day 75. Yale New Haven Health’s practice is to use fluconazole in all patients unless they have a history of a mold infection. For patients receiving tacrolimus/sirolimus, antifungal prophylaxis is not recommended due to significant azole drug interactions. The goal of this study is to compare outcomes in patients who receive fluconazole prophylaxis versus those who did not receive antifungal prophylaxis.

**Methods:**

This is a retrospective review of aHSCT patients from January 1, 2015 to July 1, 2022. Exclusion criteria were prior history of IFI requiring anti-mold treatment, concurrent prolonged neutropenia, death prior to day 75 from non-fungal etiologies or < 18 years old. Infections were diagnosed based on EORTC criteria. A subgroup analysis was performed in patients who did not receive any steroids prior to day 75. These patients were then stratified by conditioning regimen and IFI risk factors. The primary endpoint is overall breakthrough IFI rate to day 75. Secondary endpoint is the rate of IFI stratified by IFI risk group.

**Results:**

439 patients underwent aHSCT and 94 patients were excluded. Of the 345 patients included, 7 (2%) had breakthrough IFIs prior to day 75. All 7 IFIs occurred in patients who were not receiving high dose steroids. See table 1 for IFIs stratified by conditioning regimen and risk status for IFI. P values were not statistically significant. The details of the individual IFIs are further described in Table 2.

Patient Analysis

**Figure:**
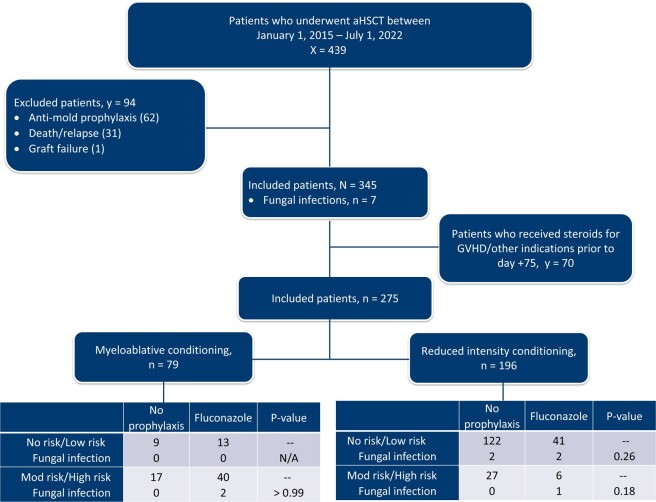

Breakthrough Fungal Infections

**Figure:**
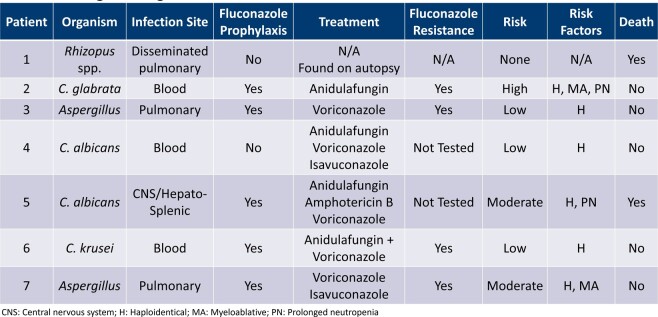

**Conclusion:**

This study revealed a low incidence of IFIs (2%) which did not correlate with the number of risk factors. Of the breakthrough IFIs, only 1 IFI could have been potentially prevented by fluconazole prophylaxis. Overall mortality was low with one patient death due to *C. albicans* who received fluconazole prophylaxis and one due to *Rhizopus spp*. Due to the low incidence of IFIs in the absence of corticosteroid use, the use of antifungal prophylaxis in aHSCT patients up to day 75 should be re-evaluated in future studies.

**Disclosures:**

**All Authors**: No reported disclosures

